# The Importance of Sleep Fragmentation on the Hemodynamic Dipping in Obstructive Sleep Apnea Patients

**DOI:** 10.3389/fphys.2020.00104

**Published:** 2020-03-13

**Authors:** Richard Staats, Inês Barros, Dina Fernandes, Dina Grencho, Cátia Reis, Filipa Matos, João Valença, João Marôco, António Bugalho de Almeida, Cristina Bárbara

**Affiliations:** ^1^Departamento do Tórax, Centro Hospitalar Universitário Lisboa Norte, Lisbon, Portugal; ^2^Instituto de Saúde Ambiental (ISAMB), Faculdade de Medicina, Universidade de Lisboa, Lisbon, Portugal; ^3^CENC - Sleep Medicine Center, Lisbon, Portugal; ^4^Faculdade de Medicina, Universidade de Lisboa, Lisbon, Portugal; ^5^William James Centre for Research, ISPA-IU, Lisbon, Portugal

**Keywords:** sleep disordered breathing, cardiovascular risk, sleep disturbance, arterial blood pressure, stroke volume

## Abstract

**Introduction:**

Obstructive sleep apnea (OSA) has been associated with non-dipping blood pressure (BP). The precise mechanism is still under investigation, but repetitive oxygen desaturation and arousal induced sleep fragmentation are considered the main contributors.

**Methods:**

We analyzed beat-to-beat measurements of hemodynamic parameters (HPs) during a 25-min period of wake–sleep transition. Differences in the mean HP values for heart rate (HR), systolic BP (SBP), and stroke volume (SV) during wake and sleep and their standard deviations (SDs) were compared between 34 controls (C) and 22 OSA patients. The Student’s *t*-test for independent samples and the effect size by Cohen’s *d* (d) were calculated. HP evolution was investigated by plotting the measured HP values against each consecutive pulse wave. After a simple regression analysis, the calculated coefficient beta (SCB) was used to indicate the HP evolution. We furthermore explored by a hierarchical block regression which variables increased the prediction for the SCB: model 1 BMI and age, model 2 + apnea/hypopnea index (AHI), and model 3 + arousal index (AI).

**Results:**

Between the two groups, the SBP increased in OSA and decreased in C resulting in a significant difference (*p* = 0.001; *d* = 0.92). The SV demonstrated a similar development (*p* = 0.047; *d* = 0.56). The wake/sleep variation of the HP measured by the SD was higher in the OSA group—HR: *p* < 0.001; *d* = 1.2; SBP: *p* = 0.001; *d* = 0.94; and SV: *p* = 0.005; *d* = 0.82. The hierarchical regression analysis of the SCB demonstrated in SBP that the addition of AI to AHI resulted in Δ*R*^2^: +0.163 and ΔF + 13.257 (*p* = 0.001) and for SV Δ*R*^2^: +0.07 and ΔF 4.83 (*p* = 0.003). The AI but not the AHI remained statistically significant in the regression analysis model 3—SBP: β = 0.717, *p* = 0.001; SV: β = 0.469, *p* = 0.033.

**Conclusion:**

In this study, we demonstrated that in OSA, the physiological dipping in SBP and SV decreased, and the variation of all investigated parameters increased. Hierarchical regression analysis indicates that the addition of the AI to BMI, age, and AHI increases the prediction of the HP evolution following sleep onset for both SBP and SV and may be the most important variable.

## Introduction

Sleep and sleep disturbances affect various components of the human homeostasis including the cardiovascular system (Muller et al., 1989; Silvani, 2019). In the last decades, the relationship between obstructive sleep apnea (OSA) and cardiovascular diseases (CVDs) has been intensively investigated by both basic science and clinical researchers (Floras, 2018). The assembled scientific evidence indicates a relevant negative impact of OSA on several components of the cardiovascular system, including an increased risk for arterial hypertension (AHT) (Javaheri et al., 2017). However, the precise mechanism of this interaction remains unclear, and the effect of OSA therapy on AHT is inconsistent. Although several longitudinal studies confirmed OSA as a risk factor for AHT and a benefit from therapy with continuous positive airway pressure (CPAP) (Peppard et al., 2000; Marin et al., 2012; Mokhlesi et al., 2014), others were inconclusive (McEvoy et al., 2016). The importance to use the correct methods for OSA diagnostic to avoid false-negative results has been recently underlined (Parati et al., 2019).

Sleep onset is usually accompanied by a physiological reduction or dipping of the blood pressure (BP) reaching about 10–20% of the values recorded during daytime (Staessen et al., 1997). A decrease in this physiological BP change is alleged to induce relevant health consequences due to an increased risk for CVD (Cuspidi et al., 2018). Compared to the daytime BP, the nocturnal BP appears more relevant for the prediction of cardiovascular risk (Fagard et al., 2008; Investigators et al., 2014). Nocturnal dipping can be reduced, absent, or even inversed by various internal and external factors including sleep disorders, obesity, high salt intake, chronic kidney disease, diabetic neuropathy, and old age (Parati et al., 2014). Although the presence and extent of dipping is very variable, OSA has been identified in several studies as a risk factor for non-dipping BP (Suzuki et al., 1996; Hla et al., 2008; McEvoy et al., 2016). Recently, the European Society of Cardiology/European Society of Hypertension considered the suspicion of nocturnal hypertension in OSA patients an indication for ambulatory BP measurement (ABPM) rather than home BP measurement (HBPM) (Williams et al., 2018). However, during the nocturnal period, the ABPM devices commonly measure the BP in fixed intervals of 30 min. Thus, the critical change of BP reduction at the wake/sleep transition is frequently missed. In fact, ABPM recordings give little detailed information over the night-time period, since both micro- and macrostructure of the sleep are not recorded. This is of clinical relevance since respiratory events during REM sleep are associated with higher BP surges (Sasaki et al., 2018) and are thought to be especially relevant for the cardiovascular risk of OSA patients (Mokhlesi et al., 2015; Mokhlesi and Varga, 2018; Varga and Mokhlesi, 2019). Missing BP recordings during REM periods due to large intervals might therefore generate nocturnal BP values that might not reflect reality. A more detailed measurement of BP values in association with sleep recordings appears therefore important to objectively assess the relationship between AHT and OSA.

At present, repetitive oxygen desaturations and activation of the sympathetic nervous system are considered the main pathomechanisms in the development of AHT in OSA (Lesske et al., 1997; Iturriaga et al., 2017). However, AHT is also linked to other sleep disorders without effect on the arterial oxygen saturation (Gottlieb et al., 2006; Abbott et al., 2019). A possible explanation for this is the complex interaction between the baroreceptor reflex and sleep fragmentation by micro-arousals (Silvani et al., 2015). Taylor et al. (2016) reported recently an increase in the daytime sympathetic activity due to sleep fragmentation that did not depend on the presence of obstructive apneas/hypopneas.

In this study, we investigated in a detailed beat-to-beat analysis the influence of either OSAs or sleep fragmentation on the evolution of hemodynamic parameters (HPs) including heart rate (HR), systolic BP, and stroke volume (SV).

## Materials and Methods

### Patients

A total of 60 participants were included. All of them were subsequently admitted to the sleep laboratory for the investigation of sleep-related breathing disorders. None of the participants was clinically unstable or referred a relevant not controlled disease. The use of beta-blockers was an exclusion criterion, while there was no further restriction regarding antihypertensive drugs including diuretics, angiotensin converting enzyme inhibitors, or angiotensin-II receptor blocker. Four patients were excluded due to major body movement during the wake/sleep transition, which did not allow enough quality in the hemodynamic data analysis. The final cohort consisted of 23 females and 33 males. Likewise, to other studies, OSA was considered present if the apnea/hypopnea index (AHI) was equal or higher than 15/h (Martinez-Garcia et al., 2013). Participants with an increased percentage of central sleep apneas (≥35%) or periodic leg movement (>15/h) were excluded.

The study was approved by the local Ethical Committee and informed consent was obtained from all participants.

### Polysomnographic Sleep Study

Sleep and sleep-related events were investigated via standard polysomnography (PSG) by Alice 5 (Koninklijke Philips N.V. Philips Respironics, Murrysville, PA, United States). The following parameters were recorded: F3, F4, C3, C4, O1, O2, M1, and M2. We used the standard referential montage of scalp electrodes against the contralateral mastoid electrode (e.g., C3/M2). Further parameters consisted of submental electrodes, thermistor and nasal pressure transducer for flow analysis, strain gauges to record respiratory movements, and EMG at both legs according to standard PSG procedures. Peripheral oxygen saturation was analyzed by pulse oximetry. The scoring of sleep and sleep-related events was based on the recommendation of the American Academy of Sleep Medicine from 2012 (Berry et al., 2015). Hypopneas were defined as a 30% decrease in nasal flow during 10 s with a 4% oxygen desaturation (acceptable hypopnea criteria according to the manual of American Academy of Sleep Medicine). This classification results in very high correlation between the AHI and the oxygen desaturation index (ODI), which are therefore interchangeable. Also, the respiratory effort related arousals (RERAs) were scored allowing to distinguish between hypoxic obstructive respiratory events alone and the total amount of obstructive respiratory events including the non-hypoxic one. Additionally, by applying the acceptable hypopnea classification, the correlation between AI and AHI decreases, thus allowing the use of both variables independently in the statistical analysis.

Sleep onset was defined as three subsequent epochs of stable sleep (in all recordings sleep stage N1 or N2). The 5 min of wakefulness prior to the first sleep epoch was considered as the wake period (WP). The following 20 min of sleep after sleep onset was considered as sleep period (SP).

#### Measurement of Hemodynamic Parameters

The Nexfin HD^TM^ monitor (BMEYE, Netherlands) has been developed to investigate non-invasively arterial BP (ABP), SV, cardiac output (CO), and peripheral vascular resistance (PVR). The method and its advantage against the non-invasive ambulatory ABP measurement (ABPM) in fixed intervals have been previously described (Wesseling, 1996). In short, the arterial pressure is measured in the finger arteries and consecutively reconstructed to brachial artery values. This methodology builds on the volume clamp technique as proposed by Penáz and the physiological criteria of Wesseling to calibrate the ABP measurement (Penaz et al., 1976; Wesseling, 1996). CO and SV are calculated by analyzing the pressure wave via a specific algorithm (de Jong et al., 2009). Frank and colleagues described the original concept already in 1899, which was further developed by Sagawa et al. (1990), Wesseling et al. (1993). This methodology allows a beat-to-beat analysis of the HPs described above. We used the heart reference system of the device to minimalize the impact of postural changes on the HP. In this study, the analyzed HPs were limited to HR, systolic BP (SBP), and SV.

#### Statistical Analysis of Hemodynamic Changes During the Wake/Sleep Transition

**FIGURE 1:**
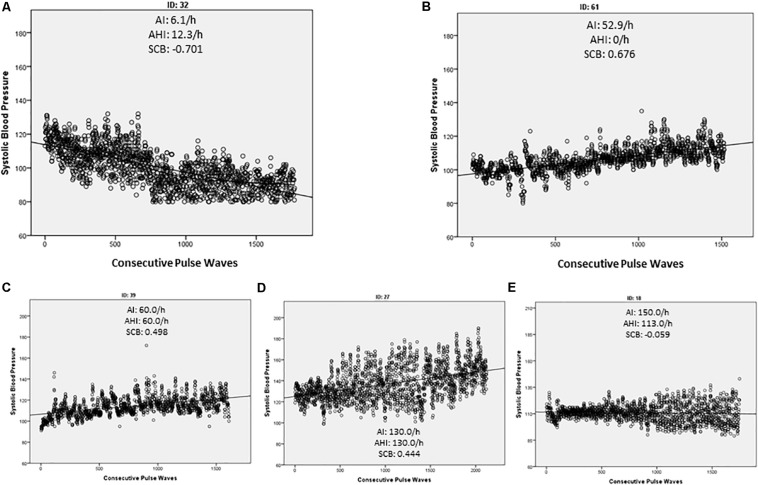
Simple dot blot of each measured pulse wave against the corresponding systolic blood pressure (SBP). The regression line indicates the general direction of the SBP during the observed time period. Arousal index (AI), apnea/hypopnea index, and the calculated standardized coefficient β (SCB) of each demonstrated example are indicated. In controls we found mainly a negative SCB indicating a decrease in the SBP **(A)** while patients with OSA tended to increase the SCB **(C,D)**. However, an increase in the SBP was also detected in the absence of a significant OSA (AHI > 15/h) if sleep fragmentation was present **(B)**. In some OSA patients, the SCB kept stable and only the variance of the SBP increased **(E)**.

Data from the Nexfin-HD device was exported and further analyzed with the SPSS Statistics software (v.24, IBM Corp., Armonk, NY, United States). Values for HR, SBP, and SV were continuously recorded over the analyzed 25-min period. For each consecutive pulse wave, the corresponding hemodynamic value was plotted in a dot blot graphic (Figure 1). A regression analysis was run with each hemodynamic value as the dependent variable and the consecutive number of the pulse wave as the independent variable. This allowed to calculate the scope of the HPs during the time window with a positive value indicating a rise and a negative value a decrease in either HR, BP, or SV. For further statistical analysis of the hemodynamic evolution at sleep onset, we utilized the standardized coefficient β (SCB).

Differences of the hemodynamic variables between wake and sleep were computed by subtraction of the average value during the WP from the average value after falling asleep. To analyze the short-term variance of the HPs, the standard deviation (SD) of each HP during sleep was subtracted from the corresponding value during wakefulness. After controlling for the normal distribution with the Shapiro–Wilk’s test (*p* > 0.05), the statistical significance between the control and the OSA group was calculated by the Student’s t-test for independent samples and the change of wake–sleep transition by the paired sample Student’s *t*-test. In case of a non-normal distribution of a variable, we applied the non-parametric Mann–Whitney *U*-test for independent samples or the related samples Wilcoxon signed-rank test, respectively.

A receiver operator characteristic (ROC) analysis was computed to investigate if the SBP values during wakefulness (WP) and sleep (SP) and the mean difference between the two periods can discriminate between the presence or absence of OSA (AHI > 15/h) or sleep fragmentation [arousal index (AI) > 15/h].

Additionally, a simple linear regression analysis was run between the consecutive heart beats or better pulse waves as the independent variable and each corresponding HP as dependent variables. The SCB was calculated and used to analyze the development of the HP during the observation period. A negative SCB is equivalent to a decrease in the investigated parameter, while a positive result indicates an increase during the SP (Figure 1).

Furthermore, a hierarchical block multiple linear regression analysis was applied to explore the impact of age, BMI, AHI, and AI during the investigated wake/sleep period, on the linear development of the HPs (increase or decrease) characterized by the SCB.

Sleep efficiency was not included since we found no significant impact on any of the investigated HPs in the initial simple regression analysis (Table 4). Model 1 consisted of age and BMI, model 2 added the AHI, and model 3 added the AI to model 2 variables. We choose to add sleep fragmentation after the AHI to investigate a possible increase in the prediction of the hemodynamic values. As stated above, the AHI and ODI were highly correlated and thus only AHI was used in this model.

A binominal logistic regression model was performed to test the prediction quality of a model including age, BMI, and AI for a non-dipping of the HP values at sleep onset. Non-dipping was classified as a SCB ≥ 0 for all three HP variables. A ROC curve was plotted to calculate the overall measure of discrimination [area under the curve (AUC)].

## Results

We found in 22 participants an AHI ≥ 15 (OSA) and in 34 an AHI < 15/h (controls). Anthropometric and polysomnographic values are listed in Table 1. Between controls and OSA, we found no statistically significant difference in either age or body mass index (BMI) (*p* > 0.05). Sleep efficiency, percentage of slow wave sleep (N3), and mean peripheral oxygen saturation (SpO2) were significantly higher in controls (both *p* < 0.05), while the AI, the AHI, and the ODI were significantly increased in the OSA group (all *p* < 0.001). There was no statistically significant difference in the percentage of oxygen saturation under 90% (T90) (*p* > 0.05). Also, no statistically significant gender difference was detected for either AHI or ODI (*p* > 0.05). Within the total study population, we found both AHI and ODI highly correlated (*r* = 0.90; *p* < 0.001). Also, for AI versus AHI (*r* = 0.80; *p* < 0.001) or AI versus ODI (*r* = 0.74; *p* < 0.001), a positive correlation was detected, although to a lower agreement compared to AHI/ODI. Thus, all three parameters were correlated with each other, but AHI and ODI could be considered almost interchangeable.

**TABLE 1:** Anthropometric and polysomnographic data.

	Age [years]	BMI [kg/m^2^]	Sleep-E [%]	N1/N2 [%]	N3 [%]	AI [/h]	AHI [/h]	ODI [/h]	Mean SpO_2_	T 90 [%]
Controls *n*: 34	44.91 (±10.0)	36.75 (±9.46)	72.6 (±17.6) *	87.53 (±19.46) *	12.46 (±19.46) *	21.51 (±20.56) **	3.08 (±5.06) **	5.76 (±14.30) **	94.85 (±1.91) *	1.65 (±6.29)
OSA *n*:22	50.41 (±10.0)	40.92 (±8.18)	62.48 (±13.06)	98.19 (±6.38)	1.80 (±6.38)	75.06 (±36.82)	66.0 (32.67)	76.35 (±34.04)	93.09 (±2.78)	11.78 (22.54)

Mean values (±standard deviation) of the anthropometric and polysomnographic data. Significant results (*p* < 0.05) are indicated by simple * and highly significant results (*p* < 0.001) with a double **. There was no statistically significant difference in age, BMI, and T 90. BMI, body mass index; Sleep-E, sleep efficiency; N1, N2 sleep stage NI or N2 or light sleep; N3, sleep stage NIII, slow wave sleep; AI, arousal index; AHI, apnea/hypopnea index; ODI, oxygen desaturation index; SpO_2_, peripheral saturation of oxygen; T90, percentage of SpO_2_ < 90%.

During the wake-to-sleep transition, almost all HPs changed significantly. The major results are listed in Table 2. Besides indicating significant results with an alpha level of 0.05, we also calculated the effect size (Cohen’s *d*) to increase the visibility of possible relevant outcomes. Controls HR and SBP decreased significantly from wakefulness to sleep, but not SV. Contrary to this result, in the OSA group, sleep onset was not associated with any significant change in HP. In both wakefulness and sleep, we detected a significantly higher HR in the OSA group. Interestingly, during wakefulness, neither the SBP nor the SV differed between the two groups. The wake–sleep transition revealed in both groups a significant change for SBP and SV. While in controls both parameters decreased, the evolution was inverse in OSA patients. The effect size of this divergent HP development was high for SBP (*d* = 0.92) and moderate for SV (*d* = 0.56). Thus, only during sleep a highly relevant difference could be seen. Contrary to this, we did not find significant results for HR (*p* = 0.49), and the effect size between the two groups was very small (*d* = 0.19).

**TABLE 2:** Student’s *t*-test and Cohen’s *d*: Hemodynamic parameters and its variation during wakefulness and sleep in controls and OSA patients.

	Controls (*SD*) (*n* = 34)	OSA (*SD*) (*n* = 22)	Δ Controls-OSAS (*SD*) significance; effect size
HR wake [/min]	67.51 (10.60)	76.93 (13.12)	−9.42 (−2.52) *p* = 0.005; *d* = 0.79
HR sleep [/min]	66.21 (11.11)	76.22 (12.01)	−10.01 (−0.90) *P* = 0.002; *d* = 0.86
Δ HR wake–sleep [/min] (*SD*); significance; effect size	1.30 (2.97); *p* = 0.016); *d* = 0.11	0.71 (3.27) *p* = 0.32; *d* = 0.07	0.59 (−0.30) *p* = 0.49, *d* = 0.19
SBP wake [mmHg]	116.82 (14.84)	123.51 (19.35)	−6.69 (−4.51) p = 0.150; d = 0.39
SBP sleep [mmHg]	113.09 (15.40)	126.78 (18.80)	−13.69 (−3.4) *p* = 0.004; *d* = 0.80
Δ SBP wake–sleep [mmHg] (*SD*); significance, effect size	3.74 (7.50); *p* = 0.007; *d* = 0.25	−3.26 (7.62) *p* = 0.058); *d* = 0.02	7.0 (−0.12) *p* = 0.001; *d* = 0.92
SV wake [ml]	100.23 (14.41)	99.20 (25.67)	1.03 (−11.26) *p* = 0.85; *d* = 0 0.05
SV sleep [ml]	98.86 (14.65)	100.75 (24.38)	−1.89 (−9.73) *p* = 0.72; *d* = 0.09
Δ SV wake–sleep [ml] (*SD*); significance, effect size	1.37 (5.52) *p* = 0.158; *d* = 0.09	−1.55 (4.78) *p* = 0.14; *d* = 0.06	2.92 (0.74) *p* = 0.047; *d* = 0.56
HR SD wake [/min]	6.40 (3.69)	5.97 (3.80)	0.43 (−0.11) *p* = 0.681; *d* = 0.11
HR SD sleep [/min]	4.69 (2.05)	7.27 (3.59)	−2.58 (−1.54) *p* < 0,001; *d* = 0.88
Δ HR SD wake–sleep [/min] (*SD*); significance; effect size	1.70 (3.07) *p* = 0.003; *d* = 0.57	−1.75 (2.67) *p* = 0.006; *d* = 0.35	3.45 (0.4) *p* < 0,001; *d* = 1.20
SBP SD wake [mmHg]	8.20 (3.26)	9.04 (2.63)	−0.83 (0.63) *p* = 0.32; *d* = 0.28
SBP SD sleep [mmHg]	7.83 (2.35)	11.73 (3.48)	−3.90 (1.13) *p* < 0.001; *d* = 1.31
Δ SBP wake–sleep [mmHg] (SD); significance; effect size	0.373 (2.89) *p* = 0.46); *d* = 0.13	−2.69 (3.51) *p* = 0.002; *d* = 0.87	3.06 (−1.86) *p* = 0.001; *d* = 0.94
SV SD wake [ml]	8.62 (4.54)	13.97 (21.53)	−5.35 (−16.99) *p* = 0.16; *d* = 0.34
SV SD sleep [ml]	7.04 (2.77)	15.32 (21.61)	−15.28 (−18.84) *p* = 0.03; *d* = 0.54
Δ SV wake–sleep SD [ml] (*SD*); significance, effect size	1.58 (3.92) *p* = 0.025; *d* = 0.42	−1.34 (3.15) *p* = 0.058; *d* = 0.06	2.92 (0.77) *p* = 0.005; *d* = 0.82

*Average (±standard deviation) of the hemodynamic values and the mean standard deviation (SD) in controls and OSA patients during wakefulness (wake) and sleep. Significance values and the effect size (Cohen’s *d*) were calculated between wakefulness and sleep for each group and between controls and OSA. Δ, difference (either wake/sleep or controls/OSA); SD, standard deviation; d, Cohen’s *d*; HR, heart rate; SBP, systolic blood pressure; SV, stroke volume.*

The assessment of the HP variability was performed by analyzing SDs of the mean values during wakefulness and sleep. In controls, sleep onset was not accompanied by any significant change in the SBP variance (*p* > 0.05). Contrary to this, we observed a significant decrease in the SD of HR and SV (*p* = 0.003 and 0.025, respectively) with a moderate effect size (*d* = 0.57 and 0.42, respectively). This reflects the physiological stabilization of the HP during sleep. In the OSA group, SD increased significantly in both HR and SBP (*p* = 0.006 and *p* = 0.002, respectively) but not in SV (*p* = 0.058). The effect size was very small for SV (*d* = 0.06), small to moderate for HR (*d* = 0.35), and high in SBP (*d* = 0.87). In the comparison of changes in the HP during sleep onset, statistically significant results were detected for all three HPs. The calculated effect size was huge for the HR exceeding one SD (*d* = 1.20) and large for SBP (*d* = 0.94) and SV (*d* = 0.82). Therefore, analyzing each group isolated, sleep onset caused only a mild to moderate change in the HP. However, since the direction was opposed (decrease in controls and increase in OSA), we found highly significant results when comparing the two groups.

To further investigate the relationship between OSA and HP during sleep, we run a receiver operator curve (ROC) analysis. SBP values discriminated between the presence and absence of OSA during sleep with an AUC of 0.745 (95% CI, 0.616–0.873; *p* < 0.001) with an overall model accuracy of 0.62. However, during wakefulness, the AUC for the SBP value was 0.596 (95% CI, 0.442–0.751), and the result did not reach statistical significance (*p* = 0.222). Since SBP during sleep revealed a discriminative capability for OSA versus non-OSA, we furthermore investigated the relevance of the change in SBP values (wake SBP values minus sleep SBV values). The AUC was 0.74 (95% CI, 0.61–0.87), indicating an acceptable discrimination. The result reached statistical significance (*p* < 0.001) with an overall model quality of 0.61. When the same analysis was run for the presence of sleep fragmentation defined as an AI > 15/h, the AUC for the SBP during sleep was 0.79 (95% CI, 0.64–0.94). On the other hand, during wakefulness, the result was not significant. The AUC for the wake–sleep difference of the SBP was 0.79 (95% CI 0.67–0.91; *p* < 0.001) and therefore good discrimination with an overall model accuracy of 0.67. The paired sample of the wake–sleep area difference under the ROC curves of the SBP reached a *z* score of −3.17 with an AUC difference of −0.154 (CI, −2.49 – 0.59), which reached statistical significance (*p* = 0.002). These results indicate that SBP values and the change of the SBP in the first 20 min of sleep discriminate between the presence or absence of OSA. This was even more evident for the presence or absence of sleep fragmentation.

Each measured pulse wave was plotted beat-to-beat against the value of the investigated HP (Figure 1). As described in the “Materials and Methods” section, the SCB was calculated to assess the continuous evaluation of the HPs during the investigated period. Controls demonstrated a negative SCB and thus a reduction in all HPs (Table 3 and Figure 1A). The effect was more visible in the SBP values compared to HR or SV. In the OSA group, the decline of the BP values was attenuated or inverted, although this was also visible in patients with sleep fragmentation without significant OSA (Figure 1B). These results reached statistical significance for SBP (*p* = 0.031) with a small to moderate effect size (*d* = 0.32) and SV (*p* = 0.033) with a medium effect size (*d* = 0.62). Results are listed in Table 3. It is of interest that we found considerable variation of the HP changes at sleep onset even in individuals with severe OSA. In Figure 1C, wake–sleep transition is accompanied by an increase in the SBP but almost without any change in variability. The participant in Figure 1D reveals an increase in both SBP and its variability. In Figure 1E, only SBP variability increases while SCB indicates a small decrease in SBP.

**TABLE 3:** Standardized coefficient β of consecutive pulse waves versus hemodynamic parameter.

	Controls *n*: 34 Average (±SD)	OSA *n*:22 Average (±SD)	*P*-value	*d*
HR SCB	−0.01 (0.28)	−0.06 (0.23)	0.483	0.19
SBP SCB	−0.16 (0.38±)	0.05 (±0.29)	0.031	0.32
SV SCB	−0.07 (±0.29)	0.09 (0.22)	0.033	0.62

*Average (standard deviation) of the standardized coefficient β (SCB), which was calculated by regression analysis of the measured hemodynamic value against each consecutive pulse wave. Controls demonstrated a negative SCB and thus decline of heart rate (HR), systolic blood pressure (SBP), and stroke volume (SV) during the 25-min period. In the OSA group, this decrease was attenuated or in case of stroke volume even inverted. The detected significance values and the effect size (*d* = Cohen’s *d*) are listed in the last two columns.*

**TABLE 4:** Simple regression analysis of anthropometric and sleep variables on the standardized coefficient β (SCB) of the hemodynamic parameters.

	SCB HR			SCB SBP			SCB SV		
			
	B (CI 95)	β	*P*	B (CI 95)	β	*P*	B (CI 95)	β	*P*
Age [years]	−0.007 (−0.014 to 0.000)*	−0.268	0.046	0.008 (−0.001 to 0.018)	0.242	n.s.	0.002 (−0.006 to 0.009)	0.07	n.s.
BMI [kg/m^2^]	−0.001 (−0.008 to 0.008)	−0.021	n.s.	−0.005 (−0.016 to 0.005)	−0.147	n.s.	−0.005 (−0.013 to 0.003)	0.162	n.s.
AHI [/h)	−0.001 (−0.003 to 0.001)	−0.194	n.s.	0.003 (0.00 to 0.005)	0.296	0.027	0.003 (0.001–0.005)	0.347	0.005
AI [/h)	−0.001 (−0.003 to 0.001)	−0.106	n.s.	0.004 (0.002 to 0.007)	0.476	0.007	0.003 (0.002 to 0.005)	0.451	<0.001
Sleep-E [%]	0.001 (−0.003 to 0.005)	0.064	n.s.	−0.002 (−0.008 to 0.004)	−0.079	n.s.	0.000 (−0.004 to 0.005)	0.023	n.s.

*Simple correlation body mass index (BMI); apnea/hypopnea index (AHI), arousal index (AI), and sleep efficiency (Sleep-E) with standardized coefficient β (SCB) of heart rate (HR), systolic blood pressure (SBP), and stroke volume (SV). The *p*-values for the statistically significant results are displayed in the third column of each hemodynamic parameter. Age negatively correlated with the HR evolution, but statistical significance was low. Both AHI and AI were positively correlated with an increase in the SBP and SV.*

To investigate the effect of anthropometric and sleep variables on the progression of the HPs, we performed a simple regression analysis with HR, SBP, and SV as dependent variables and age, BMI, AHI, AI, and sleep efficiency (Sleep-E) as independent variables. The main results are illustrated in Table 4. Sleep-E was ruled out for further analysis since no significant result was detected. Age reached statistical significance for HR and just exceeded the alpha level for SBP (*p* = 0.073). BMI did not reach statistical significance for any of the three investigated HPs, but due to its correlation with the AHI (*r*:0.256; *p* = 0.057), it was kept for further analysis.

A hierarchical multiple regression analysis was run to investigate if prediction of hemodynamic development measured by the SCB was improved by adding to age and weight (model 1) the AHI (model 2) and the AI (model 3). There was independence of residuals with the Durbin–Watson statistic of 2.337 for HR, 2.00 for SBP, and 1.60 for SV, respectively. Multicollinearity was ruled out as assessed by tolerance values above 0.2 (with a minimum of 0.297 for AHI).

For HR, none of the models increased significantly the prediction of the SCB. When analyzing the SCB of SBP, all three models reached statistical significance in the regression ANOVA analysis. However, the full model 3 of age, BMI, AHI, and AI eventually was the most robust one with *R*^2^ = 0.373; *F*(4.51) = 7.572, *p* < 0.001; adjusted *R*^2^ = 0.323. The addition of the AI to the prediction model 2 (age, BMI, and AHI) resulted in a significant increase in *R*^2^ by 0.163 and *F*(1.51) of 13.257 (*p* = 0.001). For SV, we found models 2 and 3 reaching statistical significance. For model 2 with an *R*^2^ = 0.194; *F*(3.52) = 4.16, *p* = 0.01; adjusted *R*^2^ = 0.147, the *R*^2^ increased 0.149; *F*(1/52) 9.599. In model 3, *R*^2^ reached a value of 0.206; *F*(4.51) 4.56, *p* = 0.003 and adjusted *R*^2^ = 0.206. *R*^2^ increased by 0.07 with *F*(1.51) 4.83, *p* = 0.033. Thus, the impact of the AI on the SCB of the SV was less clear compared to the results seen for SBP. The main results are listed in Tables 5–7.

**TABLE 5:** Hierarchical regression analysis of anthropometric and sleep variables for the hemodynamic standardized coefficient β prediction of heart rate.

	Standardized coefficient β (SCB) of Heart Rate (HR)					
	
Variable	Model 1		Model 2		Model 3	
			
	B (95% CI)	β	B (95% CI)	β	B (95% CI)	β
Constant	0.237 (−0.143 to 0.617)		0.202 (−0.081 to 0.585)		0.166 (−0.239 to 0.571)	
Age	−0.008 (−0.015 to 0.000)	−0.271	−0.007 (−0.014 to 0.00)	−0.271	−0.007 (−0.014 to 0.001)	0.264
BMI	0.002 (0.006 to 0.011)	0.083	−0.001 (−0.005 to 0.00)	0.115	0.004 (−0.005 to 0.012)	0.124
AHI			−0.001 (−0.003 to 0.001)	−0.162	−0.002 (−0.005 to 0.001)	0.138
AI					0.001 (−0.002 to 0.004)	0.138

	**Model 1**		**Model 2**		**Model 3**	

*R*^2^	0.078		0.319		0.328	
*F*	2.236 (n.s.)		1.962 (n.s.)		1.54 (n.s.)	
Δ*R*^2^			0.024		0.006	
Δ*F*			1.383 (n.s.)		0.348 (n.s.)	

*Hierarchical regression analysis of the SCB from heart rate. None of the models reached statistical significance. BMI, body mass index; AHI, apnea/hypopnea index; AI, arousal index; Sleep-E, sleep efficiency.*

**TABLE 6:** Hierarchical regression analysis of anthropometric and sleep variables for the hemodynamic standardized coefficient β prediction of systolic blood pressure.

	Standardized coefficient β (SCB) of Systolic Blood Pressure (SBP)					
	
Variable	Model 1		Model 2		Model 3	
			
	B (95% CI)	β	B (95% CI)	β	B (95% CI)	β
Constant	−0.229 (−0.737 to 0.280)		−0.136 (−0.628 to 0.357)		−0.392 (−0.857 to 0.073)	
Age	0.012 (0.002 to 0.021) *	0.334	0.010 (0.001-0.019	0.284	0.011 (0.003 to 0.020) *	0.321
BMI	−0.010 (−0.021 to 0.000)	−0.264	−0.013 (−0.024 to −0.002) *	−0.327	−0.011 (−0.021 to −0.001) *	−0.279
AHI			0.003 (0.001 to 0.006) *	0.314	−0.003 (−0.007 to 0.001)	−0.295
AI					0.007 (0.003 to 0.010) *	0.717

	**Model 1**		**Model 2**		**Model 3**	

*R*^2^	0.345		0.458		0.373	
*F*	3.59 (*p* = 0.034)		4,595 (*p* = 0.006)		7.572 (*p* < 0.001)	
Δ*R*^2^			0.09		0.163	
Δ*F*			5.935 (*p* = 0.018)		13.257 (*p* = 0.001)	

Hierarchical regression analysis of the SCB of systolic blood pressure. In model 1 age reached statistical significance (*p* = 0.019). In model 2 age (*p* = 0.038), BMI (*p* = 0.018), and AHI (*p* = 0.018) were statistically significant. There was a significant change compared to model 1. In model 3, age (*p* = 0.010), BMI (*p* = 0.026), and arousal index (*p* = 0.001) were statistically significant. *R*^2^*F*(1.51) increased significantly from model 2 to model 3. *p < 0.05.

**TABLE 7:** Hierarchical regression analysis of anthropometric and sleep variables for the hemodynamic standardized coefficient β prediction of stroke volume.

	Standardized coefficient β (SCB) of Stroke Volume (SV)					
	
Variable	Model 1		Model 2		Model 3	
			
	B (95% CI)	β	B (95% CI)	β	B (95% CI)	β
Constant	0.58 (−0.352 to 0.469)		0.151 (−0.235 to 0.536)		0.021 (−0.37 to 0.412)	
Age	0.004 (−0.004 to 0.012)	0.144	0.002 (−0.005 to 0.010)	0.081	0.003 (−0.004 to 0.01)	0.105
BMI	−0.007 (−0.015 to 0.002)	−0.213	−0.009 (−0.017 to −0.001) *	−0.294	−0.008 (−0.016 to 0.00)	−0.263
AHI			0.003 (0.001 to 0.005) *	0.404	0.0004 (−0.003 to 0.003)	0.005
AI					0.003 (0.00 to 0.007) *	0.469

	**Model 1**		**Model 2**		**Model 3**	

*R*^2^	0.045		0.194		0.263	
*F*	1.240 (n.s.)		4.161 (*p* = 0.01)		4.558 (*p* = 0.003)	
Δ*R*^2^			0.149		0.07	
Δ*F*			9.599 (*p* = 0.003)		4.83 (*p* = 0.033)	

Hierarchical regression analysis of the SCB of stroke volume. Both BMI and AHI reached statistical significance in model 2 (*p* = 0.035 and *p* = 0.003, respectively). *R*^2^*F*(1.52) changed significantly when compared to model 1. In model 3, only the arousal index remained statistically significant (*p* = 0.033), and we found a significant increase in *R*^2^*F*(1,51) when compared to model 2. *p < 0.05.

To confirm our assumption that AHI and ODI are interchangeable in this analysis, we recalculated model 3 with either the respiratory disturbance index (RDI) or ODI instead of AHI. As shown in Supplementary Table 1, in both cases, only the AI was significant.

**FIGURE 2:**
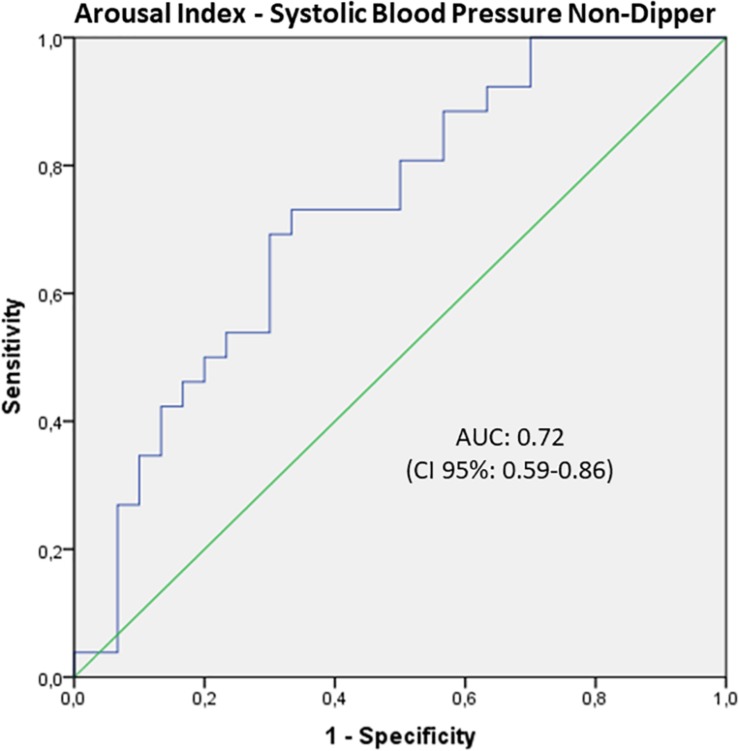
ROC curve of the arousal index to separate dipping and non-dipping systolic blood pressure (SBP). There was an acceptable discrimination by the AUC of 0.72 (95%CI: 0.59–0.86), which indicates that sleep fragmentation can be used to identify patients with non-dipping blood pressure and therefore cardiovascular risk.

Furthermore, a binominal logistic regression analysis was run to investigate the capability of a model with age, BMI, AHI, and AI to predict non-dipping of HP, defined as SCB ≥ 0. We found no statistically significant results for HR. Non-dipping in SBP was detected in 46% of the study population and therefore higher than the overall percentage of OSA patients (39.28%). The regression model was statistically significant [χ^2^(4) = 29.91; *p* < 0.001; Nagelkerke *R*^2^ = 0.54]. All four parameters reached significant results with lower values for BMI (*B* = −0.103, *SE* = 0.52; *p* = 0.045) and AHI (*B* = −0.048, *SE* = 0.02; *P* = 0.03) and higher for age (*B* = 0.170, *SE* = 0.05; *p* = 0.001) and AI (*B* = 0.042; *SE* = 0.02; *p* = 0.010). The odds ratio was 1.19 (95% CI: 1.07–1.31) for age, 0.90 for BMI (95% CI: 0.81–1.00), 1.073 for AI (95% CI: 1.02–1.12), and 0.95 for AHI (95% CI: 0.91–0.99), respectively. The overall correct prediction for the non-dipping of SBP was 78.6% with a sensitivity to identify non-dipper of 76.9% and a specificity of 80%. The positive predictive value was 66.7% with a negative predictive value of 76.9%. The ROC curve for the total model demonstrated an excellent AUC of 0.88 (95% CI: 0.78–0.97). When running the analysis only with the AI, the AUC reached a value of 0.72 (95% CI: 0.59–0.86), which can be considered an acceptable discrimination (Figure 2).

Regarding SV, we that found that, in 51.8% of the total population, the SCB of SV was equal to or superior to 0, indicating non-dipping. The regression model for non-dipping in SV reached statistical significance [χ^2^(4) = 11.19; *p* < 0.025, Nagelkerke *R*^2^ = 0.242]. However, only the BMI was a statistically significant (*p* = 0.048) predictor in the model.

## Discussion

In this study, we investigated the impact of OSA on the HPs during the wake–sleep transition. Conventionally, changes of BP from wake to sleep are investigated comparing the averages of selected periods within each state. We used this method to confirm our study protocol, but our main objective was to evaluate the impact of respiratory events and sleep fragmentation on the evolution of the recorded HP at sleep onset. This approach allows a more precise knowledge of short-term hemodynamic consequences of OSA at sleep onset and the effect of sleep disruption by respiratory events. As a major finding, we could detect that sleep fragmentation by arousals correlated better with the SBP evolution at sleep onset than the AHI.

Khatri and Freis. (1967) were one of the early groups to describe a decrease in ABP at sleep onset and a reduction in the CO due to a decreased HR. However, in this study, ABP was investigated by an intra-arterial catheter. Due to its invasiveness, this method has been, in general, replaced by a beat-to-beat measurement via the volume clamp methodology (Marrone and Bonsignore, 2018). We used the Nexfin-HD monitor in this study, which has been thoroughly investigated in several clinical settings (Fischer et al., 2012; Bubenek-Turconi et al., 2013; Pouwels et al., 2017).

When compared to the study of Khatri and Freis. (1967), our control group demonstrated a similar development of the HP. The averages between wake and sleep period demonstrated a significant decrease in HR, while the SV remained unchanged. The reduction in the SBP has been previously described by other groups and is linked to a reduction in the sympathetic-nerve activity (Somers et al., 1993). It is notable, that in our control group neither HR nor SBP reached the described reduction between 10 to 20% compared to wakefulness indicated by several authors (Silvani and Dampney, 2013 #4419; Cuspidi et al., 2018 #4505) including a joint recommendation of the International Society for Chronobiology (ISC), American Association of Medical Chronobiology and Chronotherapeutics (AAMCC), Spanish Society of Applied Chronobiology, Chronotherapy, and Vascular Risk (SECAC), Spanish Society of Atherosclerosis (SEA), and Romanian Society of Internal Medicine (RSIM) (International Society for Chronobiology et al., 2013 #4522). However, in Khatri’s study, the mean arterial pressure also reached a maximum reduction of only 8.5% when analyzed during slow-wave sleep (Khatri and Freis., 1967). Positional changes of the arm might be considered a risk factor for unreliable ABP measurement by the volume-clamp method. Leroy and colleagues found in a study with a Finapres volume-clamp device that, in fact, hand movements had little relevance on the final ABP results (Leroy et al., 1996). The more modern Nexfin-HD monitor automatically smoothens the impact of hydrostatic variation in the ABP pressure due to positional changes by means of an integrated heart reference system. Nevertheless, we excluded four participants due to large body movements and therefore a decrease in the reliability of the ABP measurement. The measured HP averages in our control group at wake–sleep transition are in accordance with other studies supporting the adequacy of our method.

A blunted hemodynamic response to sleep onset is considered a risk factor for CVDs (Hermida et al., 2013). We could show that no significant wake–sleep variation of HR, SBP, or SV occurred in the OSA group. The relevance of OSA for the existence of non-dipping ABP was recently described in the Wisconsin Sleep Cohort Study. Mokhlesi et al. (2015) found in OSA patients an increased risk of 2.84 (95% CI 1.10–7.29) for non-dipping ABP in REM sleep when compared to the control group. Our study was not designed to evaluate the overall dipping of the hemodynamic values during sleep. Nevertheless, it might be of interest that the detected effect size of OSA was moderate in case of the SV and large for SBP. These results support therefore the observation that the presence of OSA has an important impact on the non-dipping of HP during sleep. In this study, the ROC curve analysis revealed that SBP values during sleep and the wake–sleep difference of the mean SBP had acceptable discriminating capability to identify OSA patients. However, SBP was an even better discriminator for the presence of sleep fragmentation even at a relatively low value of 15 arousals/h of sleep. A missing reduction of the SBP is associated with an increase in the total cardiovascular risk (Seif et al., 2014) and can cause preclinical cardiac damage (Cuspidi et al., 2018). Therefore, our study underlines the importance to diagnose OSA in an early state of the disease to avoid possible cardiovascular side effects, which can be prevented by adequate therapy (Sapina-Beltran et al., 2019).

An increased ABP variability is considered a risk for target organ damage (Stamatelopoulos et al., 2010). In an early study, Leroy et al. (1996) demonstrated a higher ABP variation in OSA patients when compared to controls. Narkiewicz et al. (1998) reproduced this result even during wakefulness. In our study population, ABP variation during wakefulness did not differ statistically significantly between the two investigated groups. Contrary to this, with sleep onset, the variation of all HP values decreased in the control group, while OSA patients manifested the opposite reaction. Besides reaching statistical significance, the calculated effect size between the two groups was either large (SV) or very large (HR and SBP) confirming the relevance of this result.

It is difficult to compare the results between beat-to-beat and ambulatory BP measurements (ABPMs). Evidence indicates that besides the increased short-term BP variability, the midterm ABP variability investigated by ABPM is also increased in OSA (Martynowicz et al., 2016). Ke et al. (2017) associated OSA-related enhanced SBP variability in ABPM recordings with an increased risk of CVDs. In our study, the short-term HP variability was the most significant difference between controls and OSA patients. Indeed, in some OSA patients, the HP variability was the only value that changed following sleep onset. It can be concluded that OSA has, at least during sleep, a negative impact on the variation of HP. Nevertheless, a cause–effect relationship regarding CVDs needs to be further established.

To our knowledge, we are the first to investigate the influence of sleep on the HP progress by the SCB. The advantage of this method is that a successive evolution of the HP is investigated and not only a selected representative episode. Hence, the physiological progress of the HP at sleep onset is better reflected, and short periods of wakefulness or body movement have, if at all, only little influence on the results. The SCB mirrored the results detected from the sleep/wake period analysis. Controls displayed negative SCB values corresponding to the physiological decrease of HP during sleep. The OSA group exhibited an attenuation of the SCB value for SBP and reached a positive value for the SV. This reflects the impact of OSAs on the two HPs. The HR was, in agreement with the previously discussed results, not significantly different between the two groups.

Interestingly, both the simple and the hierarchical regression analyses revealed for sleep fragmentation a higher relevance concerning the ABP evolution than the AHI itself. The impact of arousals on ABP changes has been intensively investigated in previous studies. Already Somers et al. (1995) showed that OSA-induced arousals caused significant changes in the ABP. Morgan et al. (1996) found that cortical arousals induced by auditory stimuli induces a cardiovascular response leading to an increase in the ABP and HR and a decrease in the CO. Several other forms of arousals without relevant flow limitation and/or oxygen desaturations can induce transitory ABP swings (Ali et al., 1991; Davies et al., 1993; Lofaso et al., 1998). Thus, the stimulation of the central nervous system visible as acceleration in the EEG frequency induces a direct and instantaneous reaction of the cardiovascular system. Ringler et al. (1990) described that the mean arterial BP (MAP) reacted equally to hypoxic apneas as to apneas without desaturations due to oxygen supplementation. Also, acoustic arousals triggered the same hemodynamic response as hypoxic apneas, while chronic hypoxia at 80% did not induce any change in the MAP (Ringler et al., 1990). At present, it is still under discussion if arousals without respiratory events especially without relevant change in the oxygen tension can provoke a longer lasting increase in the SBP. Morrell et al. (2000) described in a cross-sectional analysis of the Wisconsin Sleep Cohort study that non-apneic participants with increased sleep fragmentation index revealed an elevated ABP when compared to controls. Stradling et al. (2000) investigated if sleep variables could predict the evening and morning ABP difference. In this study, only the ODI and the respiratory effort demonstrated a relevant independent impact on the decrease in the evening/morning ABP difference. ABP arousals measured by the pulse transient time (PTT) did not reach statistical significance, while the respiratory effort and ODI together explained 7–10% of the ABP variation (Stradling et al., 2000). We investigated the SCB of the HP instead of the evening/morning difference over a shorter time period but with continuous measurement. Nevertheless, model 2 of the hierarchical regression analysis including age, BMI, and AHI accounted for 21% variation of the systolic ABP SCB variation. By addition of the AI, we found a significant increase in the predictive value of the model to 37.3% (Table 6). In accordance to our results, Carrington and colleagues could show that artificial sleep fragmentation during 20 min after sleep onset reduced the physiological ABP decrease (Carrington and Trinder, 2008). In another study, Tayler et al. (2016) investigated the impact of several sleep parameters on the daytime sympathetic activity. They found that the sympathetic discharges correlated best with the AI followed by the ODI and discussed if sleep fragmentation by any cause could increase the probability of cardiovascular events (Taylor et al., 2016). Noda et al. (2000) detected in an ABPM study that movement arousals were the most important factor for elevated 24-h ABP values followed by the ODI. It is of importance that our study was designed to use the AHI practically interchangeable with the ODI. We controlled this assumption by using the ODI instead of the AHI in the statistical analyses and found an identical outcome. Taken alone, the AHI increases significantly the predictive value compared to age and BMI alone. Only when the AI was joined in the model 3 AHI lost its relevance in the regression model (Table 6). Sleep fragmentation due to increased respiratory effort seems unlikely to explain this result, since the substitution of RDI instead of AHI did not reveal a different result.

The relationship between SBP and sleep fragmentation was also confirmed in the ROC analysis of the SBP during the wake and sleep period. The mean SBP values during the SP and the difference of the mean SBP during the wake and sleep period demonstrated a good discrimination accuracy for the presence of sleep fragmentation. Recently, Chenini et al. (2019) described for a group of restless legs patients that the leg movement arousals were better associated with the presence of non-dipping BP than the restless legs index by itself. This study emphasizes that arousals induced by sleep-related respiratory or movement events are important for the investigation of non-dipping BP.

Interestingly, sleep efficiency had no impact at all in our model of the HP analysis. There is some evidence that both short sleep duration and insomnia might decrease cardiovascular health especially due to AHT (Anttalainen et al., 2018; Jarrin et al., 2018). However, our protocol only analyzed the percentage of wakefulness in the first 20 min of sleep. It is possible that the investigated period was too short to cause any important effect on the nocturnal HP.

There are some limitations in our study. First, the study population is relatively small. However, most of the studies using the beat-to-beat measurement technique and those cited above included a substantial smaller number of participants than ours. At present, only ABPM studies manage recruitments of large cohorts to investigate the relationship between OSA and nocturnal BP. Another possible confounding factor consists in the fact that the inflated cuff of the Nexfin/HD monitor could be responsible for sleep fragmentation. This might be particularly true in patients with a low arousal threshold, which are more commonly found in the control group. However, we cannot exclude the possibility that discomfort increased the BP or induced the artificial sleep fragmentation. The overall HP development in the control group is not in accordance to this hypothesis. The volume clamp method has been successfully tested in several sleep studies, and in our experience, the complaints about the cuff pressure started after a measurement period exceeding the one used in this study protocol. The definition of OSA in this study was an AHI of ≥15/h. We cannot rule out that in the control group, there exists a clinically relevant OSA with an AHI between 5 and 15 events/h sleep. Nevertheless, the aim of this study was to investigate which parameters influence the development of the HP at sleep onset, not OSA as a disease. Therefore, like in other studies, the criterion C of the international classification of sleep disorders was applied (American Academy of Medicine,, 2014; Genta-Pereira et al., 2018).

## Conclusion

In this study, we investigated in patients with OSA the evolution of three HPs: SBP, SV, and HR during sleep onset. In the OSA group, both SBP and SV increased during sleep resulting in a significant distinction compared to the control group. This finding reached a moderate to high effect size confirming the relevance of the results. HP short-term variation of the three parameters was significantly higher in OSA patients with either a large (SBP and SV) or very large (HR) effect size. In the ROC curve analysis, nocturnal SBP and wake–sleep BP difference demonstrated a higher AUC for sleep fragmentation than for OSA. Analysis of the HP evolution at sleep onset by means of the standardized correlation coefficient β (SCB) confirmed an opposed evolution pattern between controls and OSA patients with increasing SBP and SV in the latter group. The AI as a marker of sleep fragmentation was identified as the major contributor for HP evolution and the development of the non-dipping pattern of the SBP in OSA patients.

## Data Availability Statement

The datasets generated for this study are available on request to the corresponding author.

## Ethics Statement

The studies involving human participants were reviewed and approved by the Comissão de Ética do Centro Académico de Medicina de Lisboa (CAML), Lisbon, Portugal. The patients/participants provided their written informed consent to participate in this study.

## Conflict of Interest

The authors declare that the research was conducted in the absence of any commercial or financial relationships that could be construed as a potential conflict of interest.

## Author Contributions

RS was the project leader and main author. IB analyzed the data. DF and DG were involved in the data acquisition. CR, FM, and JV reviewed the data. JM gave support in statistical analysis. AA and CB were project supervisors and were involved in the interpretation of the data. All the listed authors contributed substantially to this manuscript.

## Abbreviations

ABP: arterial blood pressure
ABPM: ambulatory blood pressure measurement
AHI: apnea/hypopnea index
AHT: arterial hypertension
AI: arousal index
BP: blood pressure
CVD: cardiovascular disease
d: Cohen’s *D* or effect size
HP: hemodynamic parameters
HR: heart rate
MAP: mean arterial blood pressure
ODI: oxygen desaturation index
OSA: obstructive sleep apnea
RDI: respiratory disturbance index
SBP: systolic blood pressure
SCB: standardized correlation coefficient β
SP: sleep period
SRBD: sleep-related breathing disorders
SV: stroke volume
WP: wake period.
