# Supplementary material for: The Importance of Sleep Fragmentation on the Hemodynamic Dipping in Obstructive Sleep Apnea Patients
Source: Front Physiol. 2020 Mar 13;11:104. doi: 10.3389/fphys.2020.00104 (PMC7082422; doi:10.3389/fphys.2020.00104)
Supplement: Supplementary file 1 [file Table_1.docx]

# Supplementary material:

## Table sup. 1: Comparison of the apnea/hypopnea index, respiratory disturbance index and oxygen desaturation index in model 3 of the standardized coefficient β (SCB) analysis regarding Systolic Blood Pressure

| Standardized coefficient β(SCB) of Systolic Blood Pressure SBP) Model 3 | | | | | | | | |
| --- | --- | --- | --- | --- | --- | --- | --- | --- |
| Apnea/Hypopnea Index | | | Respiratory Disturbance Index | | | Oxygen desaturation index | | |
|  | B  (95%CI) | β |  | B  (95%CI) | β |  | B  (95%CI) | β |
| Constant | -0.392  (-0.857-0.073) |  | Constant | -0.341  (-0.886-0.038) |  | Constant | -0.424  (-0.813-0.132) |  |
| Age | 0.011 *  (0.003-0.020) | 0.321 | Age | 0.011  (0.004-0.021) | 0.12 | Age | 0.012  (0.002-0.020) | 0.335 |
| BMI | -0.011 *  -0.021- -0.001) | -0.279 | BMI | -0.012*  (-0.021- -0.002) | -0.30 | BMI | -0.012  (-0.022- -0.002) | -0.30 |
| AHI | -0.003  (-0.007-0.001) | -0.295 | RDI | -0.001  (-0.008-0.000) | -0.14 | ODI | -0.004  (-0.004-0.002) | -0.46 |
| AI | 0.007 *  (0.003-0.010) | 0.717 | AI | 0.005*  (0.001-0.004) | 0.58 | AI | 0.008 *  (0.002-0.009) | 0.88 |

Table sup. 2: Hierarchical regression analysis of the SCB of systolic blood pressure using model 3 with alternatively AHI, RDI or ODI. In none of the three respiratory parameters statistically significance was observed, while the AI remained significant. *=Significant results (p<0.05)
